# Brood success of sex-role-reversed pheasant-tailed jacanas: the effects of social polyandry, seasonality, and male mating order

**DOI:** 10.1186/s40851-024-00231-2

**Published:** 2024-04-30

**Authors:** Ya-Fu Lee, Yen-Min Kuo, Bing-Yuan Chuang, Hui-Ching Hsu, Yi-Jun Huang, Yu-Chen Su, Wen-Chen Lee

**Affiliations:** 1 Department of Life Sciences, National Cheng Kung University, 1 University Road, Tainan, 70101 Taiwan; 2 Pheasant-Tailed Jacana Ecological Education Center, Tainan, 72099 Taiwan

**Keywords:** Brood success, Mating order, Paternal investment, Pheasant-tailed Jacana, Social polyandry, Seasonality, Sex-role reversal

## Abstract

Multiple mating by avian females may increase hatching and overall brood success; however, reproductive effort and parental investment are costly, and females may be gradually depleted, with lowered outputs over time. Thus, males in social polyandry systems may differ greatly in their reproductive gains. In the present study, we investigated the reproductive outputs of social polyandrous and sex-role-reversed pheasant-tailed jacanas, *Hydrophasianus chirurgus*, to assess the effects of polyandry, seasonality, and male mating order on breeding success. Female jacanas produced multiple clutches, either by leaving two or more clutches with an individual male (22%), or by mating with two or more males (78%). The polyandrous females laid both the first and second clutches earlier and showed a breeding period more than twice as long as that of monandrous females. Both polyandry and seasonality affected the fate of a clutch, where clutches from polyandrous females and the early season had higher hatching and brood success rates, but the number of polyandrous females declined over the season. Polyandrous females not only laid more clutches and eggs, and gained more hatchlings and fledglings, but also achieved higher per-clutch outputs and hatching rates than monandrous females. In polyandry groups, males gained higher total hatchlings and fledglings, although not total clutches or eggs, than males in monandry or bi-andry groups. Moreover, males in polyandry groups achieved higher hatchlings and fledglings per clutch and higher hatching and brood success rates. In polyandry groups, the first-mating males obtained more clutches, eggs, and hatchlings; however, they did not have higher success rates, nor total fledglings and per-clutch outputs, than males who mated later. Overall, the results indicate a selective advantage of polyandry for the jacanas studied, particularly in the early breeding season. This advantage, however, differs both between the sexes and intra-sexually, suggesting strong connections with certain ecological/environmental conditions in addition to the jacanas’ own quality.

## Background

Polyandry is intriguing because of its intricate connections with the evolution of sexual conflicts and sex roles [1–3] and potential ecological implications for population viability and species conservation (e.g., [4–6]). Although seemingly widespread, it remains variable in both commonness and extent across and within taxa [1, 6]. The way polyandry is perfomred can also vary greatly among species, from multiple matings within a breeding cycle by females of social monogamy or polygyny systems (i.e., genetic polyandry) to the much rarer social mating system of classical polyandry [7–10], or even with males that may also have multiple mates (i.e., more strictly referred to as polygyandry; [10]).

Social (or classical) polyandry in birds is generally associated with sex-role reversal in both behaviors (e.g., more aggressive and/or territorial females than males) and morphology (e.g., larger-sized and brighter or more ornamented females than males), where females retain higher potential reproductive rate [11]. It is also commonly associated with certain life history traits (e.g., precocious young; [12]), and sole care of the young by the male, although which is not necessarily restricted to the social polyandry system [9, 13]. Even polyandry appears ubiquitous in many avian taxa [1], social polyandry is limited mainly to some waders of Charadriiformes, primarily those associated with shores or inland water bodies, including a few charadriids and scolopacids, jacanids, and the sole species of *Rostratula*, and the terrestrial buttonquails (*Turnix* spp.) and plains-wanderer (*Pedionomus*, [13, 14]; but see [9, 15] for few other terrestrial species). These birds collectively account for much less than 1% of the total avian species, in which social monogamy with female-only or bi-parental care prevail [13, 14]. The rarity and these unconventional sexual differences of social polyandry have long been noted and drawn much attention [16]; yet, its evolutionary emergence remains poorly understood and its reproductive lability not fully explored [9, 16].

Polyandry is thought to be more common in organisms in which genetic incompatibility is more likely and costly [3, 17, 18]. For instance, even in socially monogamous birds, multiple mating by females (i.e., extra-pair copulations) may increase hatching success and overall fertility [19]. Females may also gain other benefits from mating with multiple males (e.g., increased egg production, more protection and paternal care to females and offspring; reviewed in [20]). Evidence from a broad range of taxa indicates an overall positive correlation between reproductive success and mating access in females [21], which supports the argument that polyandry may influence the strength of competition in accessing multiple mates for both sexes [20], and greater inter-individual variance is expected for females in social polyandry with reversed sex-roles [20, 22].

In social polyandry systems, males may or may not father multiple clutches, whereas breeding females typically produce multiple broods from mating with one or more mates. Yet, avian parental investment (i.e., egg-laying) is costly for females in terms of both energy and nutrition [23], and physiological constraints or trade-offs may gradually deplete females, and thus reduce their outputs and reproductive gains [24]. In addition, temporal factors generate more variance in reproductive outputs (i.e., seasonality), and may even affect parental care and consequently the fate of offspring [25]. For instance, breeding late in the season or in less favorable periods may yield lowered chances of success [26], either due to the quality of the females, date effects, or the interactions between them (e.g., tree sparrows *Tachycineta bicolor*, [27]; mountain bluebirds *Sialia currucoides*, [28]).

The costs and benefits of multiple matings are unlikely to be equal for males and females [1]. For instance, sex-role reversal and polyandry may be favored in conditions where re-mating opportunities are low, particularly for males [29]. In birds, males may benefit from frequent copulation with their current mates to ensure the precedence of the last male sperm [30, 31]; however, the question of why a male would take on the role of being a subsequent mate of a polyandrous female remains intriguing. Particularly, since preceding males may gain a benefit by inseminating the female, and thus siring at least part of her subsequent brood before she deserts for another male [32–34]. Nevertheless, the relationship between the degree of polyandry and reproductive success has rarely been assessed in social polyandrous birds from the perspectives of both sexes [3, 20, 35]. Furthermore, it is also unknown whether polyandry has similar effects on the reproductive outcomes of males of different mating orders.

In the present study, we examined the reproductive outputs and variability of a socially polyandrous and sex-role-reversed wader, the pheasant-tailed jacana *Hydrophasianus chirgus*, by assessing the reproductive success of females engaging in polyandry to different extents and that of males of different mating orders. We firstly tested the hypothesis that access to mates limits female reproductive success in polyandrous sex-role-reversed species [20, 22], and predicted that jacanas in polyandry mating gain higher reproductive success than monandrous jacanas, whereas the reproductive success of females varies more than that of male jacanas [22]. Second, we tested the potential effects of seasonality on reproductive success and predicted that breeding earlier in the season leads to higher reproductive success [26, 27]. Finally, we tested whether male mating order affects male reproductive success, and predicted that males in larger breeding groups would gain lower mean reproductive outputs, whereas those of an earlier mating order would achieve higher reproductive success.

## Methods

### Study area and animals

Fieldwork was conducted in the Jacana Ecological Education Park (15 ha, hereafter referred to as the JEEP) in the Guantian district (23°11′04.2″N, 120°18′47.2″E, ca. 35 m above sea level) of Tainan City, southern Taiwan. The area is characterized by mild tropical weather with a mean monthly temperature that varies from approximately 18 °C in January to about 30 °C in July. It experiences an annual rainfall of approximately 1700 mm, concentrated in the plum rain and typhoon season, which extend from May to September (Central Weather Bureau 1981–2022 data). Guantian is one of the least populated districts in the rural region of Tainan and was historically a major agricultural center with a high density of tableland marsh lakes and irrigation canals. The JEEP was founded from abandoned or modified crop fields with the purpose of conserving the remnant jacanas in Taiwan, which dwindled to less than 50 individuals in late1990s [36]. Surrounding largely by crop fields, the park comprises around a dozen ponds of different shapes and sizes planted with prickly water lilies (*Euryale ferox*), tiger lotus (*Nymphaea lotus*), water snowflakes (*Nymphoides coreana*), water caltrops (*Trapa bicornis, T. quadrispinosa*), Manchurian wild rice (*Zizania latifolia*), and some minor plants such as alligator weeds (*Alternanthera philoxeroides*) and water primrose (*Ludwigia adscendens, L. taiwanensis*) in various proportions.

Pheasant-tailed jacanas (*H. chirgus,* Jacanidae; hereafter referred to as pheasant-tails) are widely distributed in the Indomalayan region between Pakistan and the Philippines. Like other species in this family, with the exception of lesser jacanas (*Microparra capensis*; [37]), pheasant-tails are known to engage in social polyandry with reversed sex roles and are territorial in breeding seasons [38]. In Taiwan, due to the loss of most of their natural lowland habitats, however, they are present only in small semi-natural or man-made wetlands with suitable aquatic plants and water caltrop or paddy fields, mainly in seven or eight discrete locations within or near Tainan City and are under legal protection [36, 39].

### Data collection

Due to the critical status of pheasant-tails, avoiding risks of disturbance of their clutches or broods, and a low re-sighting rate (ca. 6.3%, *n* = 48) from banding attempts at the JEEP (YF Lee, WC Lee, unpublished data), we concentrated more on obtaining effective observational data. We conducted nearly daily monitoring and observation of the jacanas in the pond sites of the JEEP throughout the breeding season from early April to late September, between 2019 and 2021. Brief breaks occurred no more than once a week and were no longer than 24 h, or rarely 48 h in cases of severe weather conditions such as typhoons. We used binoculars (Zeiss 10 × 30 Conquest Compact T*, Germany), telescopes (Kowa Prominar with TE-11WZ 25–60 × /30–70 × eyepiece, Japan), stopwatches, and counters throughout the course. When performing observations or monitoring, we hid behind bushes, tree branches, or bird-watching blinds located at a strategic distance from the ponds to avoid (or at least reduce) disturbing the birds. The onset and end of the breeding season were defined as the laying date of the first egg, observed or extrapolated, and the time the last clutch fledged, respectively [40].

### Breeding activity

Each day, depending on the number of jacanas present or participating in breeding as the season progressed, one or up to three crew members visited the pond sites to search for targeted individuals and any newly arrived jacanas. Searching and observation started at sunrise and lasted until approximately 30 min before sunset. We adopted a focal sampling approach [41] to target prior-selected either male or female jacanas residing at each designated pond, with the aim of observing breeding-related behaviors, including territoriality, courtship, mating, nesting, incubation, and caring for the young. Any arrivals of individuals new to the sites were recorded and, if becoming residing, observations followed.

Each targeted jacana was photographed (or filmed when possible), sketched with color marks, serially numbered with a unique code, and listed according to the site at which it was observed. The territoriality of pheasant-tails during the breeding season made individual identification considerably less difficult. Within each pond, the jacanas were identified according to their territory ranges, specific traits, and individual marks. Moreover, we distinguished the sex of the target jacanas based on their behaviors (e.g., courtship and egg-laying) and relative differences in body size. Individual identification was also aided by recognizable feather features on the head (e.g., the size and shape of the stripe on the top), the body (the size and shape of the side patch on the closed wings), and/or the plastic colored bands (Avian ID, Cornwall, England) applied to the jacanas during current or prior sampling (WC Lee unpublished data, YF Lee, unpublished data).

We alternated observations for each specific jacana at any specific pond site and among different time periods (morning: dawn ~ 10:00, midday: 10:00 ~ 14:00, afternoon: 14:00 ~ dusk). Each continuous observation period lasted at least one hour before shifting to another target, either at the same pond or a different pond. We recorded and timed the duration of all those breeding-related behaviors observed for later analysis. If a target took off or vanished from the sight field, we waited for at least 15 min for the target to return before shifting to the next target. Observations lasting less than 30 min were excluded from the analysis.

### Jacana abundance, clutch locality, and reproductive outputs

Two to three times each week, two or three of our team members additionally used the scan sampling method [41] to assess the entire study area in the early morning, particularly on the days when no focal sampling was performed. On these occasions, we counted all jacanas present at each pond, checked the status of the already present clutches, and recorded the localities of any new clutches. In each survey, we randomly selected the pond at which to start and alternated the sequential order among the ponds and routes followed in the survey. To calculate the reproductive outputs per clutch and success rates (number of fledglings survived out of the total eggs laid), we counted the total number of adults engaging in breeding, together with their respective clutches, eggs laid, hatchlings gained, and fledglings sighted up to the eighth week after hatching [38]. We acknowledge that lack of a genetic parentage assessment limits our inferences; nevertheless, we conducted the most intensive and thorough monitoring we could, and have used the most conservative data for analyses.

### Data analysis

We used the data recorded during our observations to verify each breeding group within a specific pond, including the territorial females and their mates, which were also territorial with generally recognizable boundaries. The focal sampling data and scanning survey data were used to obtain the inter-clutch intervals, mate-switch intervals, active breeding period lengths, and rates of clutch loss and re-nesting. The inter-clutch interval was defined as the time between the laying date of the first egg of a clutch and the onset of the first egg of the next clutch. Meanwhile, we counted the mate switch interval as the time between the first laying date of one mate and that of the next mate. The length of the active breeding period was estimated as the time between the first egg-laying of the first clutch and the last egg-laying of the last clutch. We calculated the clutch loss rate by dividing the number of clutches for which no eggs were successfully hatched by the total number of clutches included in the analysis. Finally, we evaluated the proportion of re-nesting males by dividing the number of replaceable nest losses by the number of nesting males [42].

We distinguished breeding females into monandrous, bi-androus, and polyandrous, and calculated their reproductive outputs accordingly by counting all clutches made by each female with either single, two, or multiple (three or more) male jacanas. Male reproductive outputs were distinguished into those that were associated with monandrous, bi-androus, or polyandrous breeding groups, and their mating order if in a polyandrous group. Reproductive outputs included eggs produced and hatchlings and fledglings gained in each identified clutch, and the hatching (number of chicks hatched out of total eggs), fledgling (number of hatchlings survived up to the 8th week out of total hatchlings), and success rates (number of hatchlings survived up to the eighth week out of total eggs laid), respectively.

Unless otherwise noted, all data reported in this study are presented as mean ± standard error (*SE*) or relative proportion (%) values. We conducted statistical tests using STATISTICA 12 (StatSoft, Tulsa, Oklahoma) or SPSS 28.0 (IBM Chicago, Illinois, USA) for Windows 10, with an alpha value of 0.05. Arcsine or square-root transformation for proportional or count data, respectively, was performed as necessary to meet the normality, and Leven’s test was used to examine the heterogeneity of the data.

We performed a multivariate analysis of variance (MANOVA) with Pillai’s *trace* values (*V*) to compare whether the onset of the first and second clutches differed among female jacanas with different levels of polyandry. We also performed MANOVA tests to examine the effects of the level of polyandry on the reproductive outputs, including the total number of clutches, eggs, hatchlings, and fledglings, of female and male jacanas, and those of males of different mating orders. For individual clutches, we used MANOVA with polyandry and seasonality as the main factors to assess their respective effects and interactions. When factor effects were detected, we used Tukey’s honestly significant difference (HSD) test to determine which means were significantly different [43]. We used a generalized linear model (GLM) with the clutch number as a continuous variable to examine the effects of polyandry on eggs produced and hatchlings and fledglings gained per clutch, as well as the hatching, fledging, and brood success rates of the females. Finally, correlation analyses were used to examine the relationship between the annual clutch loss rate and the proportion of polyandrous females.

## Results

We observed 92 jacana breeding groups over three seasons. These groups typically comprised single females (41, 29, and 22, respectively) and various numbers of male mates (1.8 ± 0.1, range: 1 ~ 4, *n* = 90) with confirmed courtship, incubation, and, if eggs hatched, chick caring by the males within the female’s territory over different time durations. A total of 167 males engaged in breeding over the three seasons (71, 55, and 41, respectively), resulting in a generally stable overall sex ratio of 1.7 ~ 1.9 males per female. These birds accounted for the majority of, or nearly all, the jacanas (mean weekly count: 68.8 ± 4.7 adult jacanas, maximum ranged: 88 ~ 116) occurring in the reserve during the breeding season.

### Female reproductive outputs

Monandrous females initiated their first (41.5 ± 3.98 days) and second (67.2 ± 6.21 days) clutches much later after the season onset than bi-androus (1st: 21.8 ± 2.43, 2nd: 40.6 ± 3.03) and polyandrous females (1st: 17.2 ± 3.78, 2nd: 32.9 ± 4.17; MANOVA, Pillai’s trace value *V* = 0.317, *F*_4, 142_ = 6.696, *p* < 0.001). Depending on the number of male mates present and the fate of each clutch, the length of the active breeding period varied (55.6 ± 3.92 days, range: 2 ~ 133 days, *n* = 91) among the females, but was nearly twice as long in polyandrous (76 ± 7.4 days, range: 36 ~ 133, *n* = 15) and bi-androus females (67.8 ± 5.1 days, range: 14 ~ 121, *n* = 41) than in monandrous females (32.5 ± 5.9 days, range: 2 ~ 110, *n* = 35).

We tracked 305 clutches to determine their fates and the reproductive outcomes of the breeding jacanas. Females on average produced multiple clutches (3.4 ± 0.2, range: 1 ~ 8, *n* = 90) either by laying two (31.4%) or multiple (25.7%) clutches with single males (*n* = 35, 22%), or mating with two (*n* = 40, 51.5%) or multiple males (*n* = 15, 26.6%). The inter-clutch interval for the same mate was about 28.1 ± 2.4 days (range: 3 ~ 104 days, *n* = 72), while the mate-switch intervals was 19.0 ± 1.1 days (range: 1 ~ 71 days, *n* = 144). The inter-clutch interval was positively correlated (*r* = 0.90, *p* < 0.05) with the number of adult male jacanas present, whereas the mate-switch interval was weakly negatively correlated (*r* = -0.36, *p* < 0.05) with the adult sex ratio (ASR), during the prime breeding season over the three years.

**Fig. 1:**
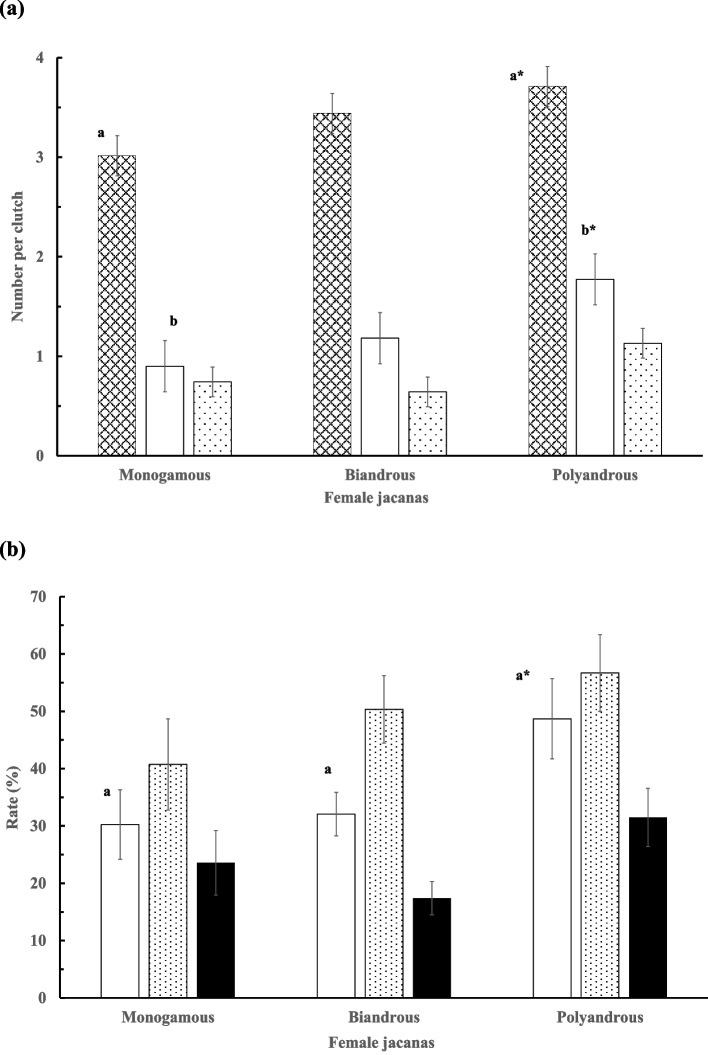
Mean (± *SE*) (**a**) egg (**▓**), hatchling (□), and fledgling (░) numbers per clutch, and (**b**) hatching (□), fledging (░), and success (■) rates (%) gained, by the monogamous, bi-androus, and polyandrous female jacanas. A letter with an asterisk indicates a significant difference in value than other values with the same letter under the same column category. ^*^p < 0.05

We found no among-year difference (*V* = 0.12, *F*_8, 170_ = 1.39, *p* = 0.201) in the total number of clutch laid and total eggs, hatchlings, and fledglings gained by each female. Females that mated with multiple males laid more clutches in a season than those with just two mates, and further more than females pairing with single mates. Consequently, polyandrous females had more eggs, hatchlings, and fledglings and had lowered coefficient of variation (CV) than bi-androus females and even more so than monandrous females (*V* = 0.68, *F*_8, 170_ = 10.95, *p* < 0.001; Table 1). In addition, polyandrous females showed higher reproductive outputs (eggs and hatchlings) per clutch (GLM; *V* = 0.341, *F*_12, 161_ = 2.802, *p* = 0.002; Fig. 1a) and a higher mean hatching rate (Fig. 1b) than monandrous females. The clutch number, however, had no effects on the reproductive outputs (*V* = 0.062, *F*_6, 81_ = 0.887, *p* = 0.51).

**Table 1:** Mean (± *SE*) clutch number and gross reproductive outputs gained by female jacanas engaging in monandrous, bi-androus, and polyandrous mating. The coefficient of variation (CV; %) of each parameter is given in parentheses underneath for each respective category. Superscript letters and asterisks indicate a significantly larger value than other values with the same superscript letter under the same column category

Female mating	Clutch	Egg	Hatchling	Fledgling
monandrous (*n* = 35)	1.91 ± 0.18^a^	5.71 ± 0.60^c^	1.54 ± 0.32^e^	1.26 ± 0.31^ g^
	(55.76)	(61.74)	(123.12)	(147.36)
Bi-androus (*n* = 40)	3.93 ± 0.26^a*,b^	13.30 ± 0.88^c*,d^	4.25 ± 0.50^e*,f^	2.23 ± 0.32^ g^
	(42.55)	(41.68)	(73.98)	(91.31)
Polyandrous (*n* = 15)	5.4 ± 0.35^a*,b*^	19.87 ± 1.31^c*,d*^	9.0 ± 1.24^e*,f*^	5.43 ± 0.92^ g*^
	(25.04)	(25.52)	(53.45)	(64.74)
Overall	3.39 ± 0.20	11.44 ± 0.74	3.99 ± 0.42	2.43 ± 0.29
	(56.07)	(60.96)	(100.17)	(112.26)

^a^*p* < 0.001, ^b^*p* < 0.05, ^c^*p* < 0.001, ^d^*p* < 0.001, ^e^*p* < 0.005, ^f^*p* < 0.001, ^g^*p* < 0.001

### Individual clutches

**Fig. 2:**
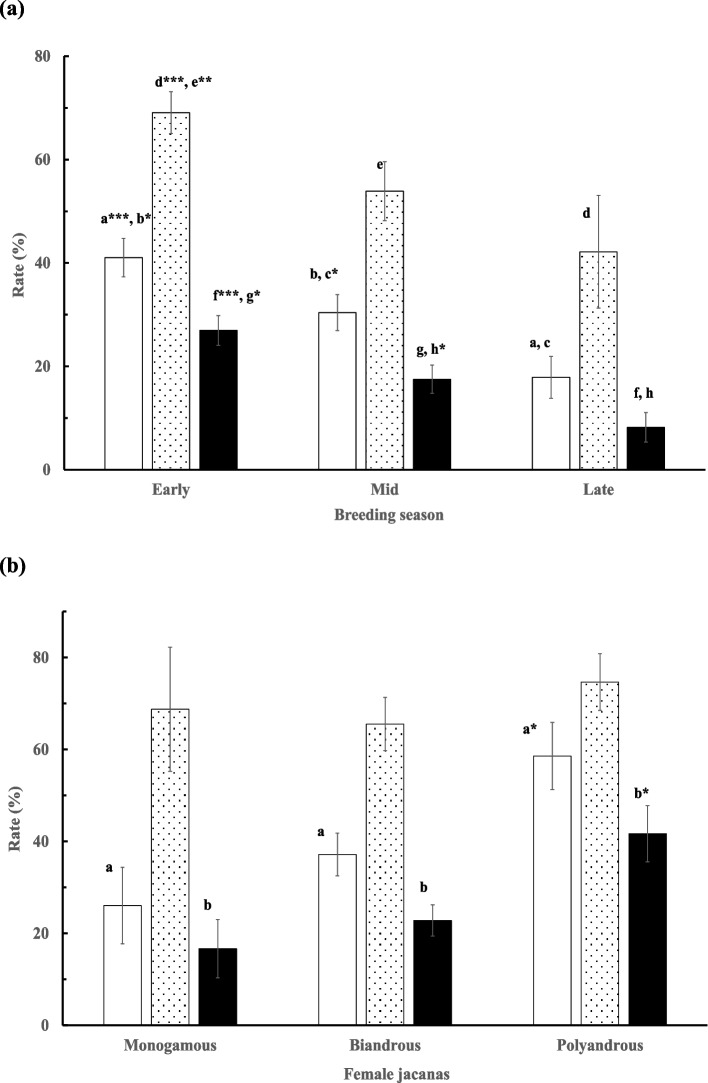
Mean (± *SE*) hatching (□), fledging (░), and success (■) rates (%) in (**a**) the early, mid, and late stages of the breeding season, and (**b**) in the early breeding season by the monogamous, bi-androus, and polyandrous female jacanas. A letter with an asterisk indicates a significant difference in value than other values with the same letter under the same column category in comparison. ^*^p < 0.05, ^**^ *p* < 0.01, ^***^p < 0.001

**Fig. 3:**
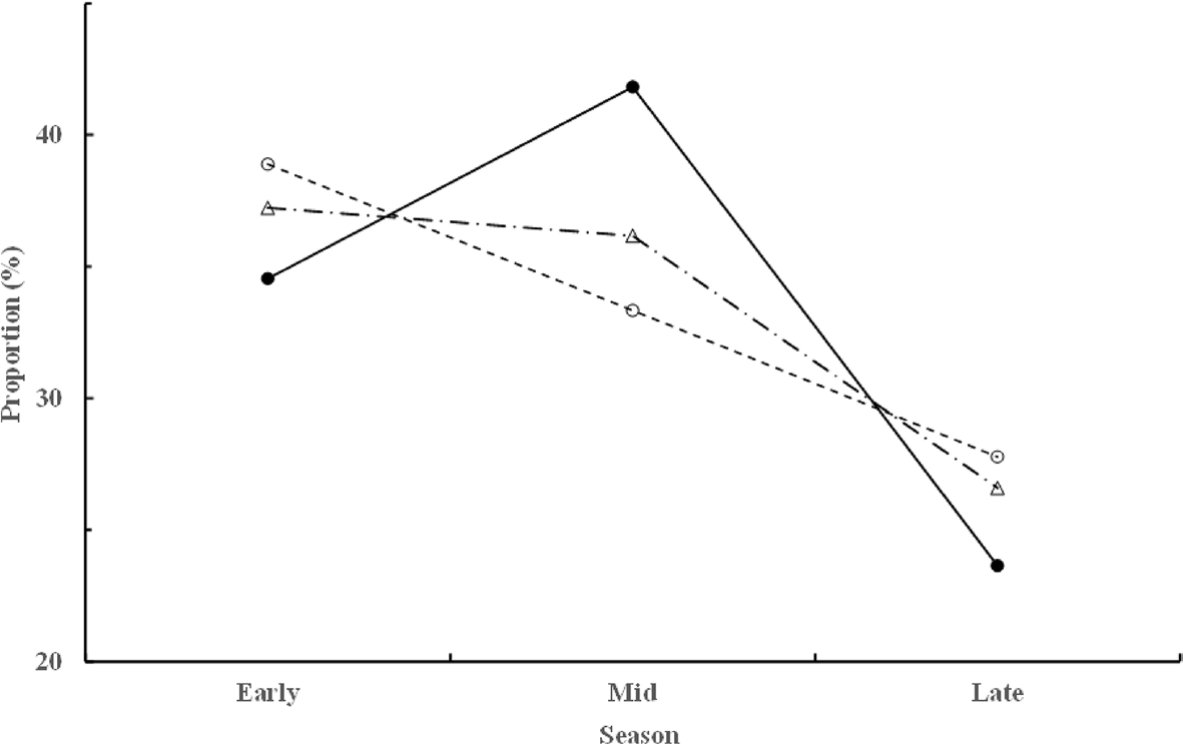
Proportions (%) of monandrous (●), bi-androus (∆), and polyandrous (○) females out of all breeding female jacanas over the early, mid, and late breeding seasons in the JEEP reserve

Both the extent of polyandry (*V* = 0.055, *F*_6, 612_ = 2.868, *p* = 0.009) and the season (*V* = 0.071, *F*_6, 612_ = 3.732, *p* = 0.001) affected the fate of the individual clutches; however, no interaction effect was observed (*p* = 0.07). Clutches from the early season (the first one-third period) had higher hatching, fledging, and clutch success rates than those laid in the mid- and late-seasons (Fig. 2a). Clutches from polyandrous females had higher hatching and clutch success rates than those from monandrous or bi-androus females (Fig. 2b). The numbers of bi-androus and polyandrous clutches generally declined, whereas those of monandrous clutches fluctuated, as the season progressed (Fig. 3; *G*_adj_ = 0.77, d.f. = 4, *p* > 0.05). Overall, the annual clutch loss rate was only weakly correlated (*r* = 0.204, *p* < 0.05) with the extent of polyandry.

### Male reproductive outputs

**Fig. 4:**
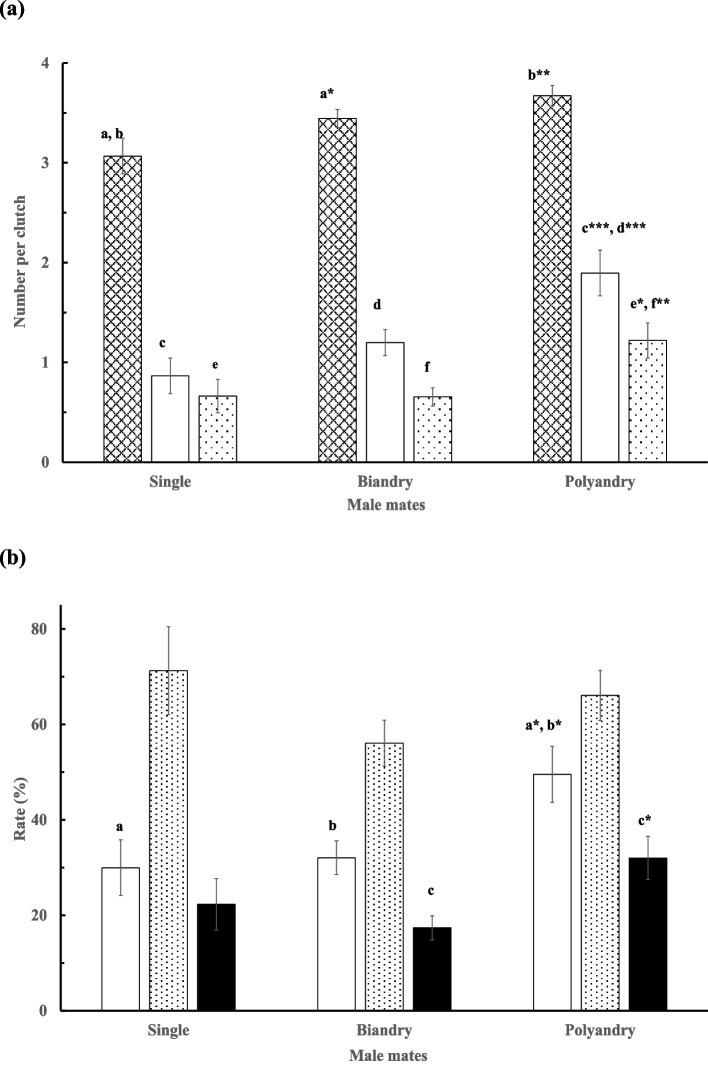
Mean (± *SE*) (**a**) clutch size (**▓**) and number of hatchlings (□) and fledglings (░) gained, and (**b**) hatching (□), fledging (░), and success (■) rates (%) achieved, by males that were the single or one of the multiple mates of bi-androus or polyandrous female jacanas. A letter with an asterisk indicates a significant difference in value than other values with the same letter under the same column category in comparison. ^*^p < 0.05, ^**^ *p* < 0.01, ^***^p < 0.001

The male jacanas did not differ in the total clutch and egg numbers gained, irrespective of whether they were single mates or one of the mates of bi-androus or polyandrous females (Table 2). Mates of monandrous females had a higher re-nesting rate (59.5%; *χ*^2^ = 18.52, *d.f*. = 2, *p* < 0.001) than that of bi-androus (55%) or polyandrous females (55.3%). However, the mates of polyandrous females gained more hatchlings or fledglings than single mates or mates in bi-androus breeding groups, respectively, and a lowered variation (*V* = 0.119, *F*_8, 318_ = 2.503, *p* = 0.012; Table 2). These mates of polyandrous females also achieved higher mean hatchlings and fledglings per clutch (*V* = 0.137, *F*_6, 320_ = 3.933, *p* < 0.001; Fig. 4a), and higher hatching and brood success rates (*V* = 0.09, *F*_6, 320_ = 2.166, *p* = 0.022; Fig. 4b).

**Table 2:** Mean (± *SE*) clutch number and gross reproductive outputs gained by the males as the single or one of the multiple bi-androus or polyandrous mates of the female jacanas. The coefficient of variation (CV, %) of each parameter is given in parentheses underneath for each respective category. Superscript letters and asterisks indicate a significantly larger value than other values with the same superscript letter under the same column category

Mate	Clutch	Egg	Hatchling	Fledgling
Monandrous (*n* = 37)	1.95 ± 0.17	5.84 ± 0.57	1.57 ± 0.31^a^	1.19 ± 0.30
	(52.72)	(59.41)	(121.73)	(153.32)
Bi-androus (*n* = 80)	1.95 ± 0.13	6.55 ± 0.44	2.11 ± 0.24	1.11 ± 0.15^b^
	(58.21)	(60.09)	(100.77)	(119.66)
Polyandrous (*n* = 47)	1.72 ± 0.11	6.19 ± 0.44	2.83 ± 0.32^a*^	1.83 ± 0.22^b*^
	(44.79)	(48.29)	(77.74)	(83.99)
Overall	1.88 ± 0.08	6.29 ± 0.28	2.20 ± 0.17	1.34 ± 0.12
	(54.01)	(56.84)	(97.44)	(115.02)

^a^*p* < 0.05, ^b^*p* < 0.05

Male jacanas in polyandrous mating groups also differed slightly in their reproductive outputs and success, depending on their order of mating. First-mated males had a high re-nesting rate (62.5%; range: 48.8 ~ 83.1 annually), nearly 2.8 times higher than that of the second- and subsequent-mated males combined (22.6%; range: 17.1 ~ 29.6 annually). Thus, the first-mated males had a higher mean total number of clutches, eggs laid, and hatchlings, but not fledglings, than subsequent male mates (*V* = 0.20, *F*_8, 244_ = 3.391, *p* < 0.005; Table 3). The reproductive outputs per clutch (*V* = 0.035, *F*_6, 246_ = 0.736, *p* = 0.62) and the mean hatching, fledging, and reproductive success rates (*V* = 0.068, *F*_6, 246_ = 1.446, *p* = 0.198) were unaffected by the mating orders.

**Table 3:** Mean (± *SE*) clutch number and gross reproductive outputs gained by the males of different mating orders among the polyandrous mates of the female jacanas. The coefficient of variation (CV, %) of each parameter is given in parentheses underneath for each respective category. Superscript letters and asterisks indicate a significantly larger value than other values with the same superscript letter under the same column category

Mate order	Clutch	Egg	Hatchling	Fledgling
First (*n* = 55)	2.29 ± 0.16^a*b*^	8.13 ± 0.55^c*d*^	2.95 ± 0.34^e*^	1.64 ± 0.21
	(51.56)	(50.12)	(85.30)	(95.08)
Second (*n* = 55)	1.60 ± 0.11^a^	5.20 ± 0.36^c^	1.95 ± 0.22^e^	1.22 ± 0.18
	(49.01)	(51.34)	(83.60)	(107.95)
Third^1^ (*n* = 17)	1.35 ± 0.12^b^	4.82 ± 0.52^d^	1.94 ± 0.55	1.06 ± 0.35
	(36.41)	(44.12)	(115.87)	(135.51)

^1^including few mates that were ranked the fourth; ^a^*p* < 0.001, ^b^*p* < 0.05, ^c^*p* < 0.001, ^d^*p* < 0.01, ^e^*p* < 0.05

## Discussion

Since Hoffmann’s (1949) earliest observations (cited in [44]), various aspects of breeding and population ecology in jacanas have been addressed. These include northern (*Jacana spinosa*; e.g., [44–47]) and wattled jacanas (*J. jacana*; e.g., [31, 47–49]) in the neotropics; African jacanas (*Actophilornis africanus*; e.g., [50]); and bronze-winged (*Metopidius indicus*; e.g., [51, 52]), comb-crested (*Irediparra gallinacean*; e.g., [53, 54]), and pheasant-tailed jacanas (e.g., [38, 55–59]) in the Indomalaya-Australasian region. The present study reports a first attempt to specifically examine the effects of the level of polyandry and seasonality on the reproductive success of both sexes and the differences between males of different mating orders for a socially polyandrous and sex-role reversed bird.

Our results showed that polyandrous female pheasant-tails had higher total reproductive outputs and gained higher success in total hatchlings and fledglings, all with lower variation, than bi-androus and monandrous females. Polyandrous females also achieved higher egg and hatchling outputs per clutch and hatching rates than monandrous females. In addition, females showed greater inter-individual variability in total reproductive outputs and gains than males. These results support our prediction that access to mates limits female reproductive success in sex-role-reversed jacanas. Along with a few other bird species and the remaining taxa in the social polyandry system (e.g., [13, 15, 16]), sex-role reversed jacanas appear to contradict the common conventional view of sexual differences in parental investments [16]. Nevertheless, our data are consistent with the parental investment theory that relative parental investment is the underlying mechanism contributing to the evolution of sex differences [9, 22].

Our results also indicated that jacana clutches laid during the early season (ca. mid-April to early or mid-June) had higher hatching, fledging, and brood success rates than those laid during the mid- and late-breeding seasons. This finding supports our prediction on seasonality and is consistent with our observation that poly- and bi-androus females laid their first and second clutches earlier than monandrous females. Earlier laying dates increase the chance of further subsequent clutches either with the same or different mates, and concur with the effects of seasonality. Birds may benefit from the seasonality effects by laying larger eggs, attaining higher hatching rates, or both (e.g., [60, 61]), owing to self or territory quality, changing environmental conditions, or both [62]. Although the ability to engage in earlier breeding may suggest certain female individual qualities [27], environmental factors (e.g., food and weather conditions) may presumably also play influential roles [27, 61, 63].

Food supply as the key of the energetic budget has a crucial effect on breeding and parental investment [9, 26, 27, 64]. Food may also enable females to lay clutches more rapidly, which is fundamental for the evolutionary rise of polyandry [9]. For instance, the level of polyandry may increase in frequency along with the habitat quality and resource abundance [65, 66]. Other life history events may also compete for energy and enhance the need for food. Temperate birds generally molt at the end of the breeding season for the coming winter (i.e., the prebasic molt), and many of them go through a complete molt that is energetically and nutritionally costly [67]. In contrast, tropical birds often tend to have protracted molting periods and involve more progressive or partial molts [68]. Pheasant-tails are primarily tropical; however, they engage in complete prebasic molt over a short period of time in late fall [38]. Thus, breeding late may expose pheasant-tails to energetic risks of overlapping the breeding costs with that of molting, which may cause slower feather growth or poorer feather condition and further affect the survival or future reproductive success [28].

Both the foliage density of the aquatic plants and the major food taken by the jacanas at the target sites generally peaked by mid-July and then declined gradually. The common food resources included water caltrop leaf beetles (*Galerucella nipponensis*, Chrysomelidae), some aquatic hemipterans (e.g., giant water bugs, Belostomatidae; backswimmers, Notonectidae), nymphs of dragonflies and damselflies, freshwater mollusks (e.g., golden apple snails *Pomacea canaliculate*, flat snail *Intha umibilicalis*; Planoridae), gobies (Gobiidae), and seeds of some aquatic plants ([36, 38, 69], YF Lee unpublished data). Seasonal changes in foliage density and food availability may also be associated with some nest predators [70], which include egrets and herons, rat snakes (*Ptyas* spp.), stripe-necked turtles (*Mauremys sinensis*), and native or exotic fishes, such as black carp (*Mylopharyngodon piceus*), walking catfish (*Clarias batrachus*) and striped snakehead (*Channa striata*) at our sites [36].

Clutches laid in the late season may expose eggs and chicks to extreme weather conditions, such as tropical cyclones (typhoons). Typhoons frequently occur in Taiwan and peak during August–September [71]. They typically bring strong winds and heavy rains, which can be lethal to eggs and hatchings due to hypothermia or being flushed away by the flooding. On the other hand, daytime temperature in the breeding season in southern Taiwan is typically warm and can become very hot as the season progresses from late spring to early summer. We observed males spending less time incubating in the morning but more time incubating at midday and during the early afternoon, presumably to prevent overheating of the eggs [69]. While heatwaves due to the warming trend may pose a significant danger to the eggs and chicks of jacanas in the summer [72], cool thermal exposure (e.g., sustained rainfall following a typhoon) and warm thermal exposure (e.g., overheating) may also negatively impact precocial young either after incubation or during incubation by delaying bone growth [63], particularly when only limited foliage coverage is available.

The patterns of male reproductive outputs resembled those of the individual clutches, even though the males differed in their breeding potentials depending on their breeding groups. Male pheasant-tails showed an overall lower variability among individuals in the number of total clutches, eggs, and hatchlings, but not fledglings, than females. Polyandry also led to fewer differences in the male reproductive gains than that of females, which concurs the prediction for social polyandry with reversed sex-roles [20]. Males in polyandry groups achieved higher total hatchlings or fledglings, although not clutches or eggs, than those in monandrous or bi-androus breeding groups. In addition, males in polyandry groups gained higher mean hatchlings and fledglings per clutch and higher hatching and brood success rates than those in monandry groups. These results help to explain, from the perspective of males’ reproductive advantage, the evolutionary emergence of social polyandry with reversed sex-roles. Our findings suggest a generally positive effect of larger polyandrous breeding groups on hatching and brood success, presumably due to the presence of two or more males engaging in parental care, even if not entirely overlapped in time, in reducing potential or actual predation risks (i.e., the many-eyes effect; [73]). Our and previous observations of onsite predators and egg/chick mortality appear to support this speculation ([38, 69], YF Lee unpublished data). Alternatively, but not mutually exclusively, it may also be that polyandrous females are less likely to flee in situations of danger and, if they do flee, may return sooner, as observed for male risk-taking behavior in polygynous birds [74]. This speculation, however, requires further analysis and investigation.

Within polyandrous groups, however, males of different mating orders differed in their reproductive outputs, wherein the first-mated males enjoyed a higher re-mating rate and the advantages of gaining more clutches, eggs, and hatchlings, but not fledglings, nor per clutch outputs or any measure in success rates, than latterly mated males. This result presumably reflects ecological (such as constant predation) and/or environmental factors that may play an influential role in determining the fate of a brood. In addition, males who engage in late breeding may have a more limited choice of partners. Such males are likely to be younger or of lower quality, or both, and perhaps cannot afford to be too selective in choosing their mates [32]. This also suggests an alternative explanation, other than a lower chance of mating, for why such males might join polyandrous females’ breeding groups rather than searching for mates among unmated females. Polyandrous females, as territory owners with evident breeding success from previous mates, may indicate certain qualities (such as age, body condition, nutritional status, and experience) to males, making them more attractive, reminiscent of female mate copying as has been observed in polygynous species [75]. However, this too is speculation and requires further investigation.

The shorter mean mate-switch interval than the inter-clutch interval suggests a connection with the availability of potential male mates, which is partially supported by the positive but weak correlation observed between the annual clutch loss rate and the level of polyandry. Rapid mate switching in sequentially polyandrous birds (e.g., red phalaropes *Phalaropus fulicarius*) results in higher levels of extra-pair paternity than in most monogamous species [76]. This may explain the occurrence of extra-pair paternity in clutches from subsequent males (the sperm storage hypothesis, [32, 77]), which creates an uneven advantage among males of different mating orders. In the present study we did not perform full-scale blood sampling for analysis, and thus we are unable to address this issue at present.

Nevertheless, extra-pair paternity is more common in simultaneously polyandrous wattled jacanas (17 ~ 29% of chicks; [31]), but rare in sequentially polyandrous comb-crested jacana (2.8%; [54]). Pheasant-tailed jacanas are also considered sequentially polyandrous [59, 69]. In addition, unlike red-necked phalaropes (*Phalaropus lobatus*, [42]) or red phalaropes [76], pheasant-tails, in common with other jacanas, are territorial, wherein both females and males defend their breeding stances [38, 69]. Our observations also indicated that male pheasant-tails may occasionally use additional measures (e.g., egg-tossing) to ensure the paternity of eggs ([52, 69], YF Lee unpublished data). These behavioral tendencies concur with the positive but weak correlation observed in the present study between the annual clutch loss rate as an indicator of male availability [42] and the extent of polyandry. Thus, the availability of males probably played only a minor or limited role as a constraint on the polyandry of pheasant-tailed jacanas at our study site.

## Conclusion

This study reveals a selective advantage in reproductive outputs and brood success enjoyed by polyandrous groups over monandrous and bi-androus groups, which is consistent between both female pheasant-tails and their mates, and particularly in the early breeding season. This advantage, however, differs subtly both between the sexes and intra-sexually, suggesting strong connections with certain ecological/environmental conditions in addition to the jacanas’ own quality. In polyandry systems, female behaviors presumably act as ultimate constraints or circumventing mechanisms for male paternity assurance by soliciting copulations from males that can care for a clutch while increasing reproductive success.

## Data Availability

Please contact the corresponding author.
